# The impact of polymyalgia rheumatica on intimate sexual relationships: findings from the PMR Cohort Study

**DOI:** 10.1093/rap/rkac070

**Published:** 2022-08-22

**Authors:** Sara Muller, Samantha L Hider, Prabath Ranasinghe, Toby Helliwell, Sarah A Lawton, William Protheroe, Christian D Mallen

**Affiliations:** School of Medicine, Keele University, Keele, UK; School of Medicine, Keele University, Keele, UK; Haywood Academic Rheumatology Centre, Midlands Partnership Foundation Trust, Stoke on Trent, UK; Health Services Office, Matale, Sri Lanka; School of Medicine, Keele University, Keele, UK; School of Medicine, Keele University, Keele, UK; School of Medicine, Keele University, Keele, UK; School of Medicine, Keele University, Keele, UK

**Keywords:** PMR, primary health care, cohort study, sexual relationships

## Abstract

**Objective:**

The aim was to determine the impact of PMR on intimate and sexual relationships over time.

**Methods:**

The PMR Cohort study is a longitudinal study of patients with incident PMR in English primary care. Participants were sent questionnaires about their PMR symptoms, treatments and overall health, including an item about how their PMR symptoms affected intimate and sexual relationships. The proportions reporting the relevance of intimate and sexual relationships, the effect of PMR on these relationships and the associations with PMR symptoms and general health were explored.

**Results:**

The baseline survey was completed by 652 of 739 patients (response 90.1%), with 446 of 576 (78.0%) responding at 2 years. The mean age of respondents was 72.4 years, and 62.2% were female. At baseline, 363 of 640 (56.7%) respondents reported that intimate and sexual relationships were not relevant to them. One hundred and thirteen of 277 (40.8%) respondents reported that PMR had a large effect on intimate relationships. This proportion decreased over time in those responding to 12- and 24-month surveys, but continued to be associated with younger age, male gender, worse PMR symptoms, poorer physical function and worse mental health.

**Conclusion:**

Intimate and sexual relationships are increasingly recognized as important for healthy ageing, and health professionals should consider this as part of a holistic approach to the management of PMR.

**Study registration:**

UKCRN ID16477.

Key messages

- PMR impacts the sexual and intimate relationships of 40% of people wanting these relationships.
- PMR–intimate and sexual relationships association was stronger in those with poorer physical and mental health.
- Clinicians should be aware of the potential effect of PMR on intimate and sexual relationships.

## Introduction

PMR is the most common inflammatory rheumatological disorder of older people. Classically, it affects people >50 years of age and is characterized by pain and stiffness in the shoulders and hips [1]. Onset can be sudden and can have a dramatic effect on activities of daily living. Although many patients respond well to treatment with glucocorticoids, PMR is a long-term condition, with treatment typically continuing for ≥2 years.

The importance of intimate and sexual relationships is increasingly being recognized as a key component of healthy ageing. Living with any long-term illness can have a significant impact on these relationships [2], with research demonstrating that intimate and sexual relationships represent an important component of quality of life and are associated with both mental and physical wellbeing [2, 3].

There is evidence for the impact of other rheumatological disorders on intimate and sexual relationships. Fatigue and ageing have been associated specifically with an impact on these relationships in people with RA [4] and AS, where 40% of people reported moderate/extreme problems with their sexual relationships [4–6]. However, there is no published evidence on the impact of PMR on intimate and sexual relationships. The aim of this study was to determine whether intimate and sexual relationships are affected by PMR symptoms in the first 2 years of the disease course and whether specific subgroups of patients are most at risk of this occurring.

## Methods

### Study design

The PMR Study is an inception cohort of people diagnosed with PMR in general practice. The study has been described in detail elsewhere [7–9]. Briefly, 739 participants were referred (between June 2012 and June 2014) to the study team by their general practitioner when a new diagnosis of PMR was made. Potential participants were sent a baseline questionnaire. Those who did not respond within 3 weeks were sent a reminder questionnaire. Response to the baseline questionnaire indicated consent to be followed up via postal survey at six further time points over 2 years (1, 4, 8, 12, 18 and 24 months), regardless of response to other follow-ups. Ethical approval for the study was received from the Staffordshire Research Ethics Committee (REC reference number: 12/WM/0021). All participants provided written informed consent.

### Data collection

The baseline survey collected information on PMR symptoms at the time of diagnosis, treatments received for PMR, general health, lifestyle, function and socio-demographics [8]. The follow-up surveys asked similar questions at six further time points over the next 2 years. Specific questionnaires in the surveys included numerical rating scales for pain and stiffness, EuroQol (EQ5D) [10], modified Health Assessment Questionnaire (mHAQ) [11, 12], Functional Assessment of Chronic Illness Therapy – Fatigue Scale (FACIT-Fatigue) [13], insomnia severity index [14], Patient Health Questionnaire (PHQ8) [15] and Generalized Anxiety Disorders (GAD7) [16].

### The impact of PMR on intimate and sexual relationships

Based on a question developed to assess the impact of AS on intimate and sexual relationships [6], participants were asked, ‘In the last **2 weeks**, how much did your PMR symptoms affect your intimate or sexual relationships?’. Response options were: ‘Does not apply to me’; ‘Not at all’; ‘A little bit’; ‘Moderately’; ‘Quite a bit’; or ‘Extremely’. This question was asked at baseline and at 12- and 24-month follow-ups.

### Statistical analyses

Simple descriptive statistics, including means, medians and percentages, as appropriate to the distribution of the data, were used to summarize patient characteristics at each time point. These statistics were then plotted and compared across responses to the intimate and sexual relationships item. All analyses were carried out in Stata v.16.2 [17] and Microsoft Excel.

## Results

### Study response

The baseline questionnaire was completed by 652 people (90.1% adjusted response), and 446 (78.0% adjusted response) people completed the 24-month follow-up. Comparison of respondents with non-respondents has been presented previously, but in summary, respondents were of higher socioeconomic status and had lower levels of pain and higher levels of general health than non-respondents and those lost to follow-up [7, 8]. The mean age of respondents at baseline was 72.4 years, and 62.2% were female.

### The impact and relevance of PMR on intimate and sexual relationships at diagnosis

**Figure 1.**
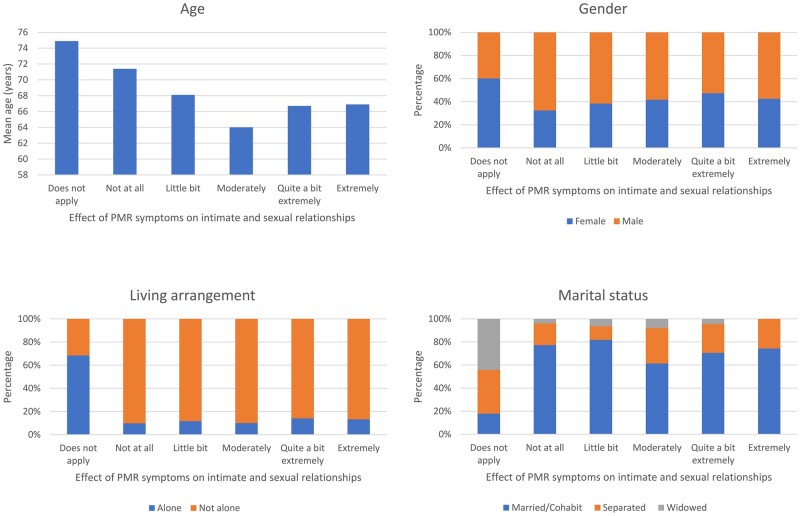
Associations between patient characteristics and reported effect of PMR on intimate and sexual relationships at diagnosis

The item regarding intimate and sexual relationships was not considered to be relevant by more than half of the cohort at baseline (*n* = 363, 56.7%) (Fig. 1). This questionnaire item was not completed by 14 people.

Older age, female gender, living alone and not being married or cohabiting were associated with reporting that intimate and sexual relationships were not relevant to an individual.

Factors associated with reporting a larger impact of PMR on intimate and sexual relationships at the time of diagnosis included lower quality of life, worse physical functioning and higher levels of fatigue, insomnia, anxiety and depression. There was much less association with pain and with stiffness severity and duration.

### The impact and relevance of PMR on intimate and sexual relationships over time

Of the 221 people reporting that intimate and sexual relationships were not relevant to them at diagnosis and responding to this question at 12- and 24-month follow-up, 177 (80%) continued to report it not to be relevant. Of the 185 for whom it was relevant at diagnosis, 20 (11%) reported that it was no longer relevant at 12 and 24 months.

### Association of PMR symptoms and health characteristics with intimate and sexual relationships at 12- and 24-month follow-up

**Figure 2.**
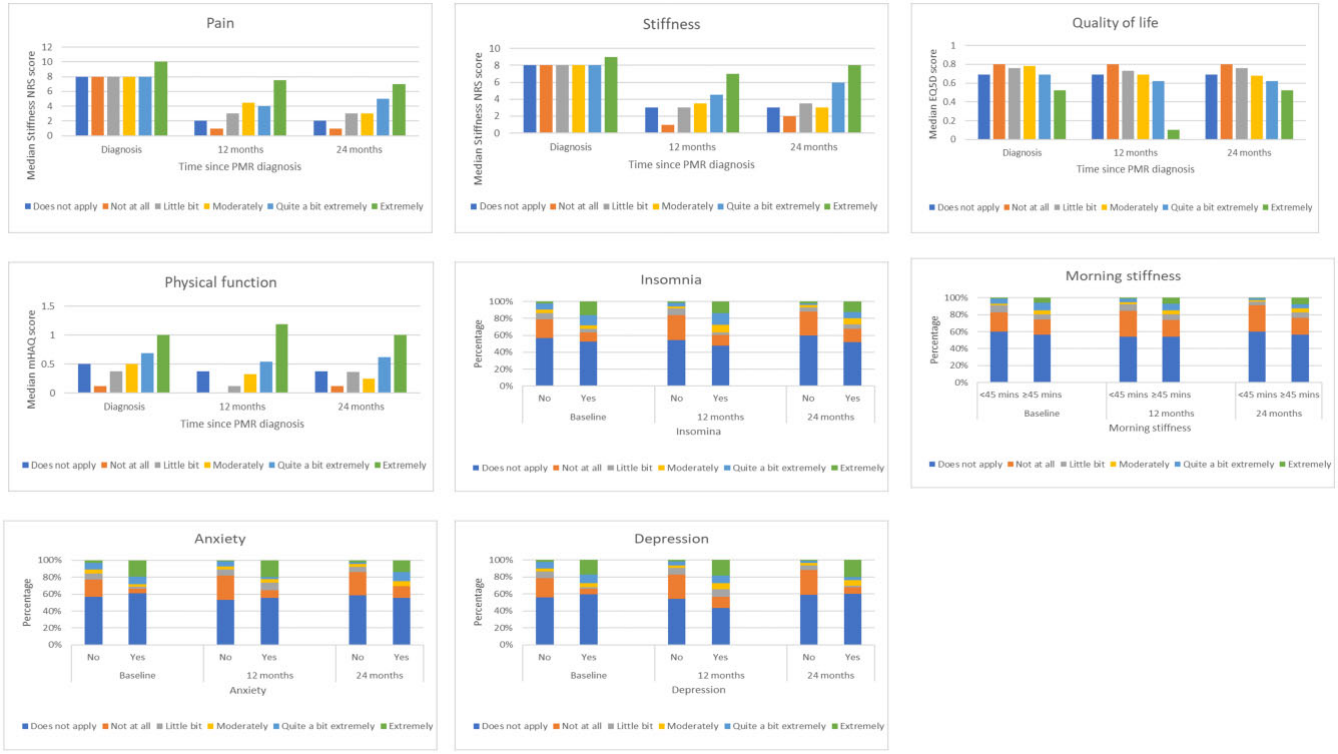
Association between concurrent patient characteristics and reported impact of PMR on intimate and sexual relationships over time

In those people who reported that intimate and sexual relationships were relevant to them at each time point, the impact of PMR on these relationships was associated with higher levels of pain and stiffness severity and duration, poorer quality of life and physical functioning, higher levels of fatigue, and with the presence of insomnia, anxiety and depression (Fig. 2). These findings were similar across time points in the study, and although improvements were seen in reported pain and stiffness at follow-ups compared with baseline, those reporting higher levels of pain and stiffness were more likely to report significant impact on intimate and sexual relationships.

## Discussion

Intimate and sexual relationships are important aspects of adult life and are increasingly being recognized as a significant aspect of healthy ageing. Interruption or reduction in the quality of such relationships can have pronounced effects, including relationship difficulties, anxiety and impacts on self-esteem and self-image [18]. In the present study, we found that almost half of people recently diagnosed with PMR say that intimate and sexual relationships are relevant to them and that two in five of these people feel that their PMR has a substantial negative impact on these relationships.

The likelihood of this key aspect of life being relevant to people with PMR was largely associated with general life circumstances, such as age, marital status and overall quality of life, that did not tend to change over time. However, in those reporting intimate and sexual relationships to be relevant, the impact of PMR on the relationship was greater in those reporting more physical and emotional symptoms. This was the case at baseline and over time. Age at diagnosis was lowest in those reporting a moderate impact of PMR symptoms on intimate and sexual relationships and higher in those reporting smaller and larger impacts.

Nicolosi *et al.* [19] found that 70% of men and 60% of women aged 40–80 years in the UK reported themselves to be sexually active. Sexual relationships therefore appear to be less relevant in our sample, but the age groups and definitions of sexual activity are not directly comparable. In those who reported being sexually active or that sexual relationships were relevant, the proportions reporting an impact were higher in the present study than in the general population in men (46% *vs* 31%), but lower in women (35% *vs* 43%), although again definitions were not directly comparable.

The frequency of PMR-related impact on intimate and sexual relationships is lower than reported estimates in RA patients (e.g. 54% of men and 46% of women [20]). This might be expected, owing to the life-long, chronic nature of RA, along with RA presenting more frequently in younger people, who are more likely to be sexually active.

A strength of this study was in recruiting participants from primary care with incident PMR and following them prospectively. This reduces the potential for recall bias and means that they are more likely to be representative of the general PMR population than cohorts recruited from specialist settings. In asking about the impact of PMR on relationships over time, we were able to gain a better understanding of how PMR is associated with intimate and sexual relationships throughout the disease course. Finally, by asking participants to report the effect of PMR on intimate and sexual relationships rather than to report sexual satisfaction, we avoided subjectivity, because people’s expectations and response to sex can vary significantly [21]. However, the item used to ask about this was taken from a previous study of people with AS [6] but has not undergone specific psychometric testing, and we allowed the study participant to define ‘intimate or sexual relationships’ as they chose. This might have introduced heterogeneity in the way participants responded, and in future studies, a more rigorously tested method of assessing impact could be used.

Although diagnosis of PMR by a general practitioner could be seen as a limitation, the sample has similar age and gender distributions to those seen in other PMR cohorts in secondary care, giving credence to diagnoses by general practitioners. Regardless of potential misdiagnosis, this sample can be seen to represent those diagnosed with and treated for PMR in real-world primary care settings. Some participants might have been referred to specialist services either at diagnosis or during treatment.

It remains unclear exactly how PMR symptoms, physical functioning and mental health are related to intimate and sexual relationships in PMR, and it is likely that, particularly for mental health, there is a bidirectional relationship. In the context of continuing PMR symptoms for many people beyond their initial diagnosis and treatment, the value in delineating these relationships further is questionable. It might be more prudent to ensure that individuals with PMR are offered a holistic approach to disease management, considering all aspects of life they consider relevant.

In conclusion, a sizeable proportion of participants reported that PMR symptoms had a significant impact on their intimate and sexual relationships, which, in turn, might have effects on physical and mental wellbeing and vice versa. The association of intimate and sexual relationships with pain and stiffness was similar to that observed in other inflammatory conditions. The continued association with symptoms and measures of function over time suggests that control of underlying disease activity could improve patients’ relationships. Our findings suggest that considering the impact on intimate and sexual relationships could form part of a broader management plan and that problems in this important area of life are likely to be related to poor symptom control, physical functioning and/or mental health.

## Data Availability

Keele University is a member of the UK Reproducibility Network and committed to the principles of the UK Concordat on Open Research Data. The School of Medicine and Keele Clinical Trials Unit have a longstanding commitment to sharing data from our studies to improve research reproducibility and to maximize benefits for patients, the wider public and the health and care system. We encourage collaboration with those who collected the data, to recognize and credit their contributions. The School of Medicine and Keele Clinical Trials Unit make data available to bona-fide researchers upon reasonable request via open or restricted access through a strictly controlled access procedure. The release of data may be subject to a data use agreement (DUA) between the Sponsor and the third party requesting the data. In the first instance, data requests and enquiries should be directed to medicine.datasharing@keele.ac.uk.
